# *Ferulagonodosa* Subsp. *geniculata* (Guss.) Troia & Raimondo from Sicily (Italy): Isolation of Essential Oil and Evaluation of Its Bioactivity

**DOI:** 10.3390/molecules25143249

**Published:** 2020-07-16

**Authors:** Natale Badalamenti, Vincenzo Ilardi, Sergio Rosselli, Maurizio Bruno, Filippo Maggi, Mariarosaria Leporini, Tiziana Falco, Monica R. Loizzo, Rosa Tundis

**Affiliations:** 1 Department of Biological, Chemical and Pharmaceutical Sciences and Technologies (STEBICEF), University of Palermo, Viale delle Scienze, ed. 17, I-90128 Palermo, Italy; natale.badalamenti@unipa.it (N.B.); maurizio.bruno@unipa.it (M.B.); 2 Department of Earth and Marine Sciences (DISTeM), University of Palermo, Via Archirafi 26, I-90128 Palermo, Italy; vincenzo.ilardi@unipa.it; 3 Department of Agricultural, Food and Forest Sciences (SAAF), University of Palermo, Viale delle Scienze, ed. 4, I-90128 Palermo, Italy; sergio.rosselli@unipa.it; 4 Centro Interdipartimentale di Ricerca “Riutilizzo Bio-Based Degli Scarti da Matrici Agroalimentari” (RIVIVE), University of Palermo, I-90128 Palermo, Italy; 5 School of Pharmacy, University of Camerino, Via Sant’Agostino 1, I-62032 Camerino, Italy; filippo.maggi@unicam.it; 6 Department of Pharmacy, Health Science and Nutrition, University of Calabria, I-87036 Arcavacata Rende (CS), Italy; mariarosarialeporini@tiscali.it (M.L.); tiziana.falco@unical.it (T.F.); rosa.tundis@unical.it (R.T.)

**Keywords:** essential oil, gas chromatography-mass spectrometry, antioxidant, diabetes, obesity

## Abstract

*Ferulago nodosa* (L.) Boiss. (Apiaceae) is a species occurring in the Balkan-Tyrrhenian area. The object of the present study is Sicilian *F. nodosa* subsp. *geniculata* (Guss.) Troia & Raimondo, classified as an endemic *F. nodosa* subspecies. Aerial parts of this plant species were subjected to hydrodistillation to obtain an essential oil. A total of 93 compounds were identified with 2,3,6-trimethyl benzaldehyde (19.0%), spathulenol (9.0%), (*E*)-caryophyllene (5.4%), and caryophyllene oxide (5.4%) as the main components. The biological activities of *F. nodosa* essential oil were also investigated. This oil showed an interesting antioxidant potential in a 2,2′-Azino-bis(3-ethylbenzothiazoline-6-sulfonic acid) (ABTS) test (IC_50_ of 14.05 μg/mL). Additionally, hypoglycemic and antilipidemic effects were evaluated. Lipase enzyme was inhibited with an IC_50_ value of 41.99 μg/mL. Obtained data demonstrated that *F. nodosa* could be considered a promising source of bioactive compounds useful for the treatment and management of obesity.

## 1. Introduction

The genus *Ferulago* W.D.J. Koch (Apiaceae) includes about 50 species distributed in the Mediterranean area, from Portugal to Iran and from Russia to Northwestern Africa [1,2].

The center of origin and diversification of the genus was identified to be in Turkey, which houses 34 species, many of which are exclusive [1,2]. Tomkovich and Pimenov [2] divide the genus *Ferulago* into two subgenera (*Ferulago* and *Galbanifera*) and nine sections.

In Sicily, the genus *Ferulago* is represented by *Ferulago nodosa* (L.) Boiss. and *Ferulago campestris* (Besser) Grecescu, both belonging to the subgenus *Galbanifera* [2]. In particular, *F. nodosa*, the object of this contribution, highlights a Balkan-Tyrrhenian distribution, being present in Greece, Albania, and probably also in the Yugoslav Republic of North Macedonia, with a western population exists in Sicily.

Based on morphological characteristics and in consideration of the current geographical and genetic isolation of the Sicilian population, Peruzzi et al. [3] proposed to assign the Sicilian population of *Ferulago nodosa* to the rank of subspecies, with the combination *Ferulago nodosa* subsp. *geniculata* (Guss.) Troia & Raimondo.

The genus *Ferulago* is recognized in traditional medicine as a remedy for gastrointestinal tract panics, ulcer. Moreover it was known as sedative, a treatment for hemorrhoids, skin infection and spleen disease, as well as a natural preservative in different foods, such as meat and cheese [4].

Oxidative stress is defined as an imbalance between the production of Radical Oxygen Species (ROS) and the antioxidant defensive mechanisms in our bodies, that results in the development of pathological conditions such as pre-diabetes [5,6]. It was reported that 5–10% of people per year with pre-diabetes would progress to diabetes and its associated complications, such as obesity [6]. Therapeutic implications of the pre-diabetes condition include the inhibition of α-amylase and α-glucosidase enzyme suppressing postprandial hyperglycemia and reduced dietary carbohydrate digestion and absorption [7]. In addition, inhibition of pancreatic lipase, with a reduction of fat absorption, is a therapeutic strategy to reduce the risk of obesity [8]. Thus, natural inhibitors of these enzymes and antioxidant agents from dietary plants may be proposed as an effective therapy for management of post-prandial hyperglycemia and obesity diseases.

Essential oils have been used since ancient times due to their biological properties. Many essential oils, in fact, are characterized by antioxidant properties [9]. For this reason, it will be used as a substitute for butylated hydroxyanisole and butylated hydroxytoluene (BHT), which are harmful to human health [9]. For this reason, essential oils from edible plants, alone or inserted in active packaging and edible coatings, may represent a valid instrument to prolong shelf life. Several research papers evidenced that *Ferulago* extracts and essential oils are characterized by antioxidant activity and are used as medicinal plants or food preservatives [10,11,12].

Moreover, several essential oils have been proven to possess hypoglycemic and hypolipidemic properties [13,14,15,16]. However, no previous study investigated the inhibition of carbohydrate-hydrolyzing enzymes and the hypolipidemic activity of *Ferulago* subspecies.

For this purpose, this study aimed to investigate the phytochemical composition of Sicilian *F. nodosa* subsp. *geniculata* essential oil obtained by the hydrodistillation of aerial parts, as well as its biological properties, including antioxidant, hypoglycemic, and hypolipidemic potentials.

## 2. Results and Discussion

### 2.1. Essential Oil

The chemical composition of the essential oil obtained from the aerial parts of *F. nodosa* subsp. *geniculata* is reported in Table 1. A total of 93 compounds, accounting for 73.9% of the total composition, were identified. From a semi-quantitative perspective, they were represented by aromatics (21.0%, shared by 5 constituents), followed by oxygenated sesquiterpenes (17.5%, 11 constituents), sesquiterpene hydrocarbons (12.4%, 21 constituents), monoterpene hydrocarbons (8.8%, 17 constituents), and oxygenated monoterpenes (8.4%, 24 constituents).

The major components were the aromatic, 2,3,6-trimethyl benzaldehyde (19.0%), and the sesquiterpenoids, spathulenol (9.0%), (E)-caryophyllene (5.4%), and caryophyllene oxide (5.4%).

Among monoterpenoids, bornyl acetate (4.6%) and α-pinene (2.9%) were the most representative compounds. The presence of aromatic aldehydes, such as 2,3,6-trimethyl benzaldehyde, is considered a hallmark of several *Ferulago* species [18,19,20,21,22,23]. It is worth mentioning that these compounds are formed during distillation from the cleavage and molecular rearrangement of ferulol-type monoterpenoids [19].

The essential oil obtained from the aerial parts of *F. nodosa* growing in Sicily has previously been studied by Ruberto et al. [18], who found a different chemical profile, with 2,3,4-trimethylbenzaldehyde (42.2%) and α-pinene (22.4%) as the main constituents. These authors did not detect the 2,3,6-trimethylbenzaldehyde which instead was the major component of our sample. Demetzos et al. [24] studied the essential oil composition of flowering aerial parts from *F. nodosa* growing in Greece and identified α-pinene (31.1%) as the major component. Notably, they did not detect any aromatic aldehydes. Similar results were published by Evergetis et al. [25], who studied the essential oil profile of *F. nodosa* growing in Greece, showing α-pinene (30.8%) and β-phellandrene (10.2%) as the main constituents. Thus, the notable chemical polymorphism observed may be the result of significant variability in genetics (e.g., occurrence of different subspecies) together with the influence of geographic factors and the type of processing and extraction of the plant material (e.g., hydrodistillation vs. steam distillation).

### 2.2. Antioxidant Activity of Essential Oil

The antioxidant potential of *F. nodosa* subsp. *geniculata* essential oil was screened using different assays (Table 2). ABTS^+^ radical cation resulted more sensitive to the action of *F. nodosa* essential oil with an IC_50_ value of 14.0 μg/mL, while an IC_50_ value of 26.3 μg/mL was observed in a 2,2-diphenyl-1-picrylhydrazyl (DPPH) assay. The results can be justified because ABTS^+^ and DPPH^.^ radicals have different stereochemistry, and after a reaction with essential oil these two radicals give diverse responses. Percentages of 39.4% and 32.7% after 30 and 60 min of incubation, respectively, were recorded in a β-carotene bleaching test when the essential oil was tested at maximum concentration. In a FRAP assay, an IC_50_ value of 16.9 μg/mL was observed.

Several research articles that have *Ferulago* ssp. as the object of investigation confirmed the antioxidant potential of these plants. Previously, Cecchini et al. [26] compared the DPPH radical scavenging activity of *F. campestris* fruit and root essential oils, and found IC_50_ mean values of 0.23 and 0.24 μg/mL of oil, respectively. In disagreement with our data, both oils were able to protect lipids from peroxidation in a similar manner to the positive control BHT (IC_50_ mean values of 0.010 and 0.011 μg/mL for fruit and root essential oils, respectively). A great variability was observed in the DPPH radical scavenging activity of *F. angulate* essential oils derived from different organs. Among them, unripe seed oil showed the highest radical scavenging potential with an IC_50_ value of 162 μg/mL, followed by leaf (IC_50_ value of 210 μg/mL). These organs are characterized by a higher phenol and flavonoid content, compared with other parts [10]. Lower DPPH radical scavenging was found for Iranian *F. angulata* aerial part essential oil (IC_50_ value of 488 μg/mL) [27]. A similar situation was observed for *F. trifida* essential oils with IC_50_ values in the range 95–120 μg/mL [28].

Our data are in line, also, with those reported by Karakaya et al. [29], who investigated the antioxidant potential of *F. pauciradiata* extracts and essential oils derived from roots and aerial parts. The highest DPPH activity was observed in root essential oil and ethyl acetate fraction, with IC_50_ values of 4.59 and 6.56 μg/mL, respectively. Shahbazi and Shavisi [30] compared the DPPH radical scavenging activity of sub-fractions of methanol extract and the essential oil obtained from *F. bernardii* aerial parts, and found IC_50_ values of 5.66, 6.88 and 14.81 mg/mL, respectively.

The aqueous and methanol extracts obtained from flowers, stems, and leaves of *F. angulata* were investigated by Faride et al. [31], who found IC_50_ values ranged from 214 to 1606 µg/mL against the DPPH radical. Values in the range from 264 to 393 μmol of Trolox equivalent per gram of dry weight were found for *F. angulata* ethanol flower extract and leaf methanol extract, respectively [32].

The antioxidant potential of this species was confirmed in vivo. In fact, treatment with *F. angulata* extract at doses from 200 to 800 mg/kg/day resulted in an increase in catalase, glutathione peroxidase, and super oxide dismutase activities in diabetic Wistar rats [33].

Mileski et al. [34] reported the antioxidant activity of methanol, ethanol, and aqueous extracts obtained from aerial parts and inflorescences of *F. macedonica*. The DPPH results demonstrated that extract derived from the inflorescences (in the range of 490–1170 μg/mL) were more active than the extract obtained from the aerial parts (630–1810 μg/mL). This evidence was confirmed, also, in an ABTS test. The antioxidant potential of the crude extract and four fractions of *F. carduchorum* aerial parts, at two vegetative stages, were analyzed by Golfakhrabadi et al. [35]. The highest DPPH radical scavenging activity was found for flower crude extract, with an IC_50_ of 0.49 mg/mL, followed by fruit crude extract (IC_50_ of 0.62 mg/mL) and flower methanol fraction (IC_50_ of 0.68 mg/mL).

Among the identified compounds, spathulenol, the main abundant volatile component of the *F. nodosa* essential oil, was able to inhibit both DPPH and ABTS radicals, with IC_50_ values of 85.60 and 639.25 μg/mL, respectively [36]. Additionally, this tricyclic sesquiterpenoid exhibited protection against lipoperoxidation in rat brains, with an IC_50_ value of 26.13 μg/mL and, consequently, decreased the generation of malondialdehyde [36]. Coté et al. [37] demonstrated the DPPH radical scavenging potential of α-pinene and caryophyllene oxide, with IC_50_ values of 3.4 and 183 μg/mL, respectively, while IC_50_ > 200 μg/mL was found for both β-caryophyllene and bornyl acetate.

### 2.3. Hypoglycemic and Hypolipidemic Potential of Essential Oil

*F. nodosa* essential oil α-amylase, α-glucosidase, and lipase inhibitory activities were concentration-dependent, and results are summarized in Table 3. The essential oil exhibited promising lipase enzyme inhibitory activity, with an IC_50_ value of 42.0 μg/mL. This value was 0.8 times higher than that found for the positive control, orlistat. A lower enzyme inhibitory activity was found against α-amylase and α-glucosidase, with IC_50_ values of 196.4 and 365.9 μg/mL, respectively.

The α-glucosidase and α-amylase inhibitory activities of *F. bracteata* root extracts were investigated by Karakaya et al. [38]. Dichloromethane and ethyl acetate extracts exhibited the lowest IC_50_ values of 0.95 μg/mL, followed by methanol extract (IC_50_ of 4.19 μg/mL), whereas the aqueous extract was inactive. The effects of *F. angulata* hydroalcoholic extract were evaluated by Musavi-Ezmareh et al. [39] in diabetic male rats. After supplementation of 200 mg/kg body weight of extract for four weeks, a reduction of blood sugar and an improvement of blood lipid profiles were observed. A similar situation was observed with supplementation of *Ferulago angulata* hydroalcoholic extract in Wistar rats, where a reduction of total cholesterol, low-density lipoproteins, and triglycerides, and an inhibition of lipid peroxidation were observed [39,40]. More recently, Parsamehr et al. [41] confirmed that intraperitoneal injection of *F. angulata* hydroalcoholic extract for three weeks in diabetic rats could be effective in the treatment of diabetes and at the same time alleviate liver damage.

Several terpenes identified in *F. nodosa* essential oil are able to exert hypoglycemic and hypolipidemic activity. The intraperitoneal administration of α-pinene in diabetic mice at different doses (0.05, 0.10, 0.25 and 0.50 mL/kg) resulted in a reduction of fasting blood glucose levels [42]. Previously, Bae et al. [43] demonstrated that intraperitoneal administration of α-pinene (5, 25, or 50 mg/kg) reduced body weight and the serum levels of α-amylase and lipase. Additionally, the combination of β-caryophyllene (500 μmol) and L-arginine (500 μmol) stabilized glucose tolerance and reduced pancreatic cell damage in diabetic rats [44].

Zhou et al. [45] suggested, also, that β-caryophyllene could be used as a therapeutic target for the treatment of diabetic patients, since this terpene acts at the level of arginine-specific mono-ADP-ribosyltransferase 1. Moreover, β-caryophyllene oral administration at a dose of 200 mg/kg induced a reduction of glucose, increase of plasma insulin levels, and improvement of altered activities of carbohydrate metabolic enzymes [46].

## 3. Materials and Methods 

### 3.1. Chemicals and Reagents

Solvents of analytical grade were purchased from Honeywell (Seelze, Germany). Reagents, α-amylase, lipase from porcine pancreas, and α-glucosidase from *Saccharomyces cerevisiae* were purchased from Sigma-Aldrich S.p.a. (Milan, Italy). Acarbose from *Actinoplanes* sp. was obtained from Serva (Heidelberg, Germany).

### 3.2. Plant Materials

Aerial parts of *F. nodosa* subsp. *geniculata* were harvested in April 2019, immediately before flowering, from different plants growing on a flat-lying litho-soil near Noto Antica, Syracuse, (Sicily, Italy) (36°57′27.37” N; 15°02′18.76” E) of 378 m above sea level. This plant has a distribution limited to the southeastern sector of Sicily, in particular the complex of the Iblei Mountains. It is a circular-limestone plateau of circular shape, dating back to the Miocene, in which the presence of numerous streams dug deep incisions (canyons), locally known as “cave iblee”. The bioclimate of the area is thermo-Mediterranean dry, which favors both aspects of thermo-xerophilous pine forest (*Pinus halepensis* and *Coridothymus capitatus*) and all aspects of mesophilic shrub (*Myrtus communis*, *Arbutus unedo*, and *Pistacia lentiscus*). Samples were identified by Prof. Vincenzo Ilardi, University of Palermo, Italy. A voucher specimen (PAL 109707) has been deposited in the Herbarium Mediterraneum Panormitanum of the “Orto Botanico”, University of Palermo, Italy.

### 3.3. Isolation of the Essential Oil

Fresh leaves and stems (200 g) of *F. nodosa* subsp. *geniculata* were reduced to small pieces and subjected to hydrodistillation according to the standard procedure previously described [47]. The obtained oil (0.22% *w*/*w*) was stored in dark vials at −20 °C before analyses.

### 3.4. GC-FID Analysis of the Essential Oil

An Agilent 4890D gas chromatograph (GC) coupled with an ionization flame detector (FID) (Santa Clara, CA, USA) was used to analyze the essential oil. The separation stationary phase was represented by an HP-5 capillary column (5% phenylmethylpolysiloxane, 25 m, 0.32 mm i.d.; 0.17 μm f.t.) (Agilent, Folsom, CA, USA). The mobile phase was helium (99.999%) flowing at 1.0 mL/min. The oven temperature programmer was as follows: 60 °C isothermal for 5 min, then ramp (4 °C/min) to 220 °C, and ramp (11 °C/min) to 280 °C. The essential oil was diluted 1:100 in hexane and the volume injected was 1 μL in split mode (1:34). The injector and detector temperatures were set to 280 °C. A commercial mix of *n*-alkanes (C_8_–C_30_) purchased from Supelco (Bellefonte, CA, USA) was used to determine the peak linear retention index (RI). Quantitative values, expressed as percentages, were obtained following the procedure of Cecchini et al. [26].

### 3.5. GC-MS Analysis of the Essential Oil

An Agilent 6890N GC equipped with a 5973N single quadrupole mass spectrometer (MS) (Santa Clara, CA, USA) was employed. The stationary phase was an HP-5MS capillary column (30 m, 0.25 mm i.d., 0.1 μm f.t.) (Agilent, Folsom, CA, USA). The operative conditions and the mobile phase were the same as those reported above. The injector and transfer line temperatures were 280 and 250 °C, respectively. The same dilution as that reported above was injected into the GC-MS system in split mode (1:50). Mass spectra were acquired in electron impact (EI) mode in the range of 29–400 *m/z*. The identification was carried out by a combination of MS matching and RI overlapping against the Adams 2007 [17], NIST 17 (NIST 17, 2017) [48], and FFNSC2 (FFNSC2) [49] libraries. The comparison with available authentic standards (Sigma-Aldrich, Milan, Italy) was also used.

### 3.6. Antioxidant Activity 

The in vitro antioxidant potential of *F. nodosa* subsp. *geniculata* essential oil samples was screened by ABTS, DPPH, β-carotene bleaching test, and FRAP assay. Both DPPH and ABTS tests were applied to examine the radical scavenging activity of the essential oil, using the procedure previously described by Loizzo et al. [50]. In both cases, ascorbic acid was used as a positive control.

In the β-carotene bleaching test a mixture of linoleic acid, β-carotene, and Tween 20 was prepared and the resulting emulsion was mixed with the essential oil at different concentrations (2.5–100 μg/mL) [50,51]. The absorbance was read after 30 min of incubation at 470 nm. Propyl gallate was used as a positive control. The essential oil at a concentration of 2.5 mg/mL was tested, also, to evaluate the ability of samples to protect iron from a redox reaction [52]. BHT was used as a control.

### 3.7. Hypoglycemic and Hypolipidemic Potential

In the α-amylase inhibitory test, a mixture of α-amylase, starch, and essential oil was prepared as previously reported [52]. In the α-glucosidase inhibitory test, a mixture of maltose, α-glucosidase, *ο*-dianisidine, peroxidase/glucose oxidase, and samples was prepared. In both cases, essential oil was tested at different concentrations, ranging from 25 to 1000 μg/mL, and absorbance was measured at 540 and 500 nm, respectively [50].

The pancreatic lipase inhibitory test was performed as previously reported [53]. A mixture of *p*- nitrophenyl caprylate and porcine pancreatic lipase enzyme was prepared and added to 96-well-plate-contained essential oil at different concentrations (2.5–40 μg/mL). After that, the absorbance was measured (405 nm).

## 4. Conclusions

The goal of this study was to chemically characterize and biologically investigate the essential oil obtained from *F. nodosa* subsp. *geniculata* aerial parts collected in Sicily (Italy). 

The major components of essential oil were 2,3,6-trimethyl benzaldehyde, spathulenol, (*E*)-caryophyllene, and caryophyllene oxide. Among monoterpenoids, bornyl acetate and α-pinene were the most representative compounds. *F. nodosa* subsp. *geniculata* essential oil showed, also, an interesting antioxidant potential, with particular reference to ABTS testing. Additionally, hypoglycemic and antilipidemic effects were evaluated with interesting activity against lipase. Obtained data demonstrated that *F. nodosa* subsp. *geniculata* essential oil could be considered a promising phytocomplex useful for formulation of nutraceutical products to prevent diseases associated with oxidative stress, such as type 2 diabetes and obesity.

## Figures and Tables

**Table 1:** Chemical composition of the essential oil of the aerial parts (leaves and stems) of *F. nodosa* subsp. *geniculata* from Sicily.

Compound	R_i_^a^	R_i_^b^	a.p. (%) ^c^
Santolina triene	904	906	0.8 ± 0.07
Tricyclene	914	921	t
*α*-Thujene	918	924	t
*α*-Pinene	923	932	2.9 ± 0.30
Camphene	936	946	0.3 ± 0.02
Sabinene	962	969	0.5 ± 0.04
*β*-Pinene	965	974	0.2 ± 0.02
Dehydro-1,8-cineole	984	988	0.1 ± 0.01
Myrcene	985	988	0.8 ± 0.07
*δ*-2-Carene	995	1001	0.2 ± 0.02
*m*-Mentha-1(7),8-diene	1000	1000	t
(*3Z*)-Hexenyl acetate	1007	1004	t
1,2,4-Trimethyl benzene	1016	1021	0.2 ± 0.02
*p*-Cymene	1019	1020	0.9 ± 0.08
Limonene + *β*-Phellandrene	1022	1024 + 1025	1.8 ± 0.11
(*Z*)-*β*-Ocimene	1034	1032	0.1 ± 0.01
*β*-Isophorone	1036	1044	0.1 ± 0.01
Benzene acetaldehyde	1040	1036	t
(*E*)-*β*-Ocimene	1043	1044	0.3 ± 0.02
γ-Terpinene	1053	1054	t
Terpinolene	1082	1086	t
*p*-Cymenene	1084	1089	t
6-Camphenone	1089	1095	0.1 ± 0.01
*α*-Pinene oxide	1090	1099	0.3 ± 0.02
Isophorone	1115	1118	0.2 ± 0.01
*α*-Campholenal	1121	1122	t
*cis*-Limonene oxide	1128	1132	t
*trans*-Pinocarveol	1131	1135	t
*trans*-*p*-Menth-2-en-1-ol	1134	1136	0.1 ± 0.01
Camphor	1136	1141	t
*trans*-Verbenol	1139	1140	0.1 ± 0.01
1,4-Dimethyl-δ-3-tetrahydroacetophenone	1145	1152	t
Borneol	1157	1165	0.5 ± 0.04
(*E*)-Isocitral	1168	1177	0.1 ± 0.01
Terpinen-4-ol	1171	1174	t
2,4-Dimethyl-benzaldehyde	1176	1178	0.6 ± 0.05
Cryptone	1179	1183	0.4 ± 0.03
*p*-Cymen-8-ol	1181	1179	0.2 ± 0.02
*cis*-Piperitol	1189	1195	0.1 ± 0.01
*trans*-Piperitol	1203	1207	0.1 ± 0.01
4-methylene-Isophorone	1209	1216	t
*β*-Cyclocitral	1214	1217	t
Cumin aldehyde	1233	1238	0.1 ± 0.01
*cis*-Chrysanthenyl acetate	1256	1261	1.0 ± 0.12
Bornyl acetate	1279	1287	4.6 ± 0.51
*trans*-Sabinyl acetate	1287	1289	0.2 ± 0.01
*trans*-Pinocarvyl acetate	1293	1298	0.2 ± 0.02
Carvacrol	1302	1298	0.2 ± 0.01
2,3,4-Trimethyl benzaldehyde	1306	1315	1.0 ± 0.11
Myrtenyl acetate	1319	1324	0.1 ± 0.01
*δ*-Elemene	1329	1335	0.3 ± 0.02
2,3,6-Trimethyl benzaldehyde	1346	1352	19.0 ± 1.89
*α*-Copaene	1364	1374	0.1 ± 0.01
*β*-Bourbonene	1372	1387	0.8 ± 0.07
*β*-Cubebene	1380	1387	0.1 ± 0.01
*β*-Elemene	1382	1389	0.7 ± 0.06
*α*-Cedrene	1396	1410	0.2 ± 0.01
(*E*)-Caryophyllene	1405	1417	5.4 ± 0.58
*β*-Copaene	1415	1430	0.2 ± 0.02
Aromadendrene	1423	1439	0.2 ± 0.02
*α*-Humulene	1437	1452	0.5 ± 0.06
*cis*-Cadina-1(6),4-diene	1450	1461	0.1 ± 0.01
*α*-Acoradiene	1451	1464	0.1 ± 0.01
*γ*-Muurolene	1463	1478	0.1 ± 0.01
Germacrene D	1465	1484	1.3 ± 0.14
*γ*-Gurjunene	1469	1475	0.1 ± 0.01
*α*-Curcumene	1472	1479	0.5 ± 0.04
(*E*)-*β*-ionone	1474	1487	0.2 ± 0.01
Bicyclogermacrene	1479	1500	0.9 ± 0.10
*α*-Muurolene	1486	1500	0.1 ± 0.01
Cuparene	1487	1504	0.1 ± 0.01
*β*-Bisabolene	1497	1505	0.3 ± 0.03
*α*-Cuprenene	1499	1505	0.3 ± 0.04
(*E*)-Nerolidol	1555	1561	0.2 ± 0.02
Spathulenol	1562	1577	9.0 ± 0.97
Caryophyllene oxide	1565	1582	5.4 ± 0.51
Viridiflorol	1574	1592	0.5 ± 0.04
Cubeban-11-ol	1577	1595	0.3 ± 0.02
Rosifoliol	1584	1600	0.6 ± 0.05
Humulene epoxide II	1590	1608	0.5 ± 0.04
*trans*-Isolongifolanone	1605	1625	0.2 ± 0.01
*epi*-*α*-Muurolol	1626	1640	0.5 ± 0.06
*α*-Cadinol	1639	1652	0.3 ± 0.02
Neophytadiene	1834	1838	0.2 ± 0.01
Phytone	1839	1843	0.2 ± 0.02
Benzyl salicylate	1852	1864	0.1 ± 0.01
Hexadecanoic acid	1961	1959	0.2 ± 0.01
Phytol	2099	2103	1.6 ± 0.18
Tricosane	2301	2300	0.1 ± 0.01
Pentacosane	2500	2500	0.1 ± 0.01
Heptacosane	2700	2700	0.2 ± 0.02
Nonacosane	2900	2900	2.5 ± 0.26
**Class of Compounds**			**±0.07**
Oxygenated Monoterpene			8.4
Monoterpene Hydrocarbons			8.8
Sesquiterpene Hydrocarbons			12.4
Oxygenated Sesquiterpene			17.5
Aromatic			21.0
Others			5.8
**Total**			**73.9**

R_i_^a^: retention index on a HP-5MS column; R_i_^b^: retention index from Adams [17]; ^c^: relative peak area ± standard deviation (S.D.) (*n* = 3); t: traces.

**Table 2:** The antioxidant potential of *F. nodosa* subsp. *geniculata*.

	DPPH Test IC_50_ (µg/mL)	ABTS Test IC_50_ (µg/mL)	β-Carotene Bleaching Test (IC_50_, µg/mL or % Inhibition)		FRAP μM Fe (II)/g
			t 30 min ^a^	t 60 min ^a^	
*Essential oil*	26.3 ± 4.3	14.0 ± 1.1	39.4%	32.7%	16.9 ± 1.3
*Positive control*					
Ascorbic acid	5.1 ± 0.82	1.7 ± 0.3			
Propyl gallate			0.09 ± 0.08	0.09 ± 0.06	
BHT					63.4 ± 4.6

Data are expressed as means ± S.D. (n = 3). ^a^: at maximum concentration tested of 100 μg/mL.

**Table 3:** Hypoglycemic and hypolipidemic potential (IC_50,_ µg/mL) of *F. nodosa* subsp. *geniculata*.

	α-Amylase	α-Glucosidase	Lipase
*Essential oil*	196.4 ± 4.3	365.9 ± 5.1	42.0 ± 2.1
*Positive control*			
Acarbose	50.6 ± 0.9		35.8 ± 1.3
Orlistat			37.4 ± 1.1

Data are expressed as means ± S.D. (n = 3).
